# Plant Volatile Analogues Strengthen Attractiveness to Insect

**DOI:** 10.1371/journal.pone.0099142

**Published:** 2014-06-09

**Authors:** Yufeng Sun, Hao Yu, Jing-Jiang Zhou, John A. Pickett, Kongming Wu

**Affiliations:** 1 State Key Laboratory for Biology of Plant Diseases and Insect Pests, Institute of Plant Protection, Chinese Academy of Agricultural Sciences, Beijing, People's Republic of China; 2 Department of Entomology, Henan Institute of Science and Technology, Xinxiang, People's Republic of China; 3 Department of Biological Chemistry and Crop Protection, Rothamsted Research, Harpenden, Hertfordshire, United Kingdom; Institute of Vegetables and Flowers, Chinese Academy of Agricultural Science, China

## Abstract

Green leaf bug *Apolygus lucorum* (Meyer-Dür) is one of the major pests in agriculture. Management of *A. lucorum* was largely achieved by using pesticides. However, the increasing population of *A. lucorum* since growing Bt cotton widely and the increased awareness of ecoenvironment and agricultural product safety makes their population-control very challenging. Therefore this study was conducted to explore a novel ecological approach, synthetic plant volatile analogues, to manage the pest. Here, plant volatile analogues were first designed and synthesized by combining the bioactive components of *β*-ionone and benzaldehyde. The stabilities of *β*-ionone, benzaldehyde and analogue **3 g** were tested. The electroantennogram (EAG) responses of *A. lucorum* adult antennae to the analogues were recorded. And the behavior assay and filed experiment were also conducted. In this study, thirteen analogues were acquired. The analogue **3 g** was demonstrated to be more stable than *β*-ionone and benzaldehyde in the environment. Many of the analogues elicited EAG responses, and the EAG response values to **3 g** remained unchanged during seven-day period. **3 g** was also demonstrated to be attractive to *A. lucorum* adults in the laboratory behavior experiment and in the field. Its attractiveness persisted longer than *β*-ionone and benzaldehyde. This indicated that **3 g** can strengthen attractiveness to insect and has potential as an attractant. Our results suggest that synthetic plant volatile analogues can strengthen attractiveness to insect. This is the first published study about synthetic plant volatile analogues that have the potential to be used in pest control. Our results will support a new ecological approach to pest control and it will be helpful to ecoenvironment and agricultural product safety.

## Introduction

The green leaf bug, *Apolygus lucorum* (Meyer-Dür) (Hemiptera: Miridae), is a major pest of many cultivated plants including cotton, cereals, vegetables and fruit trees. With the reduced application of insecticides on Bt cotton for the control of lepdopitera pests, the abundance of *A. lucorum* has substantially increased in China [1]. This species can easily attain outbreak densities, switch hosts, and wilder spread because of its environmental adaptability [1]–[4], high population growth rate [2]–[3], and strong dispersal capacity [5]–[6]. These characteristics make *A. lucorum* difficult to control.

Currently, various agronomic (soil tillage, removing weeds before sowing seeds) and chemical (spraying with organophosphates, pyrethroids and nicotinoid) measures are applied to control *A. lucorum*. To reduce large yield losses caused by the pest and insecticide use which are usually harmful to human beings and the environment, it is necessary to develop new approach in integrated pest management (IPM) schemes for this pest.

Insect ecology involves the relationship between insect and its surroundings that seek both to proceed with IPM and to protect the ecological environment [7]. Research on insect ecology is helpful to ecoenvironment and agricultural product safety. Plant volatiles, a factor of insect ecology, emitted by plant, in response to mechanical or herbivore damage, may achieve this aim and can be applied at the farm or landscape level. They involve mediating the behavior of insects [8]–[9], natural enemies [10]–[12] or neighboring plant [13]–[15]. Their potential value in pest population control has been recognized [16]–[18].

The current study concerns the use of plant volatiles, *β*-ionone and benzaldehyde, as attractants for *A. lucorum*. According to previous reports, *β*-ionone, a volatile released from cotton, tomato, and other plants [19]–[23], attracts brown planthoppers [24] and repels phytophagous mites [25]. Benzaldehyde, a common component of plant volatiles [26], attracts many pest species [27]–[28]. A recent study has indicated that *β*-ionone and benzaldehyde can be recognized by adult *A. lucorum* and can affect *A. lucorum* behavior under laboratory conditions [29]. However, the two chemicals cannot be efficiently used as *A. lucorum* attractants because of their low stability and mediocre ability to attract *A. lucorum* in field conditions.

Indeed, so far there has been no publish about plant volatile analogues synthesis and their use. In the current study, we try to explore novel ecological approach to control pest based on synthetic plant volatile analogues. Here we first hypothesized that the analogues synthesized with substructure combination strategy by combining the bioactive components of *β*-ionone and benzaldehyde would contribute to achieving our objectives: i) to increase stability by changing chemical functional groups, for example, aldehyde group, which is associated with low oxidative stability and (ii) to enhance attractiveness to *A. lucorum* by combining the active groups of the two compounds. Based on such working hypothesis, we designed and synthesized 13 analogues of *β*-ionone and benzaldehyde. We tested the stabilities of *β*-ionone, benzaldehyde and analogue **3 g**. And we then conducted EAG test, behavior assay and filed experiment to evaluate the attractivities of the analogues to *A. lucorum*.

## Materials and Methods

### Ethics statement

We captured the insects in Xinxiang Experiment Station (Henan) of Institute of Plant Protetion, Chinese Academy of Agricultural Sciences. The wild-captured *A. lucorum* used in this study was serious pest in China. Therefore, no specific permits were required for the described insect collection and experimentation.

### Insects

A culture of *A. lucorum* was maintained on fresh ears of corn (*Zea mays* L.) in a climate chamber at 29±1°C, 60±5% RH, and 14∶10 L:D at the Institute of Plant Protection, Chinese Academy of Agricultural Sciences, Beijing. Adults were used in the laboratory experiment.

### Synthesis of *β*-ionone and benzaldehyde analogues 3a−m

**Figure 1:**
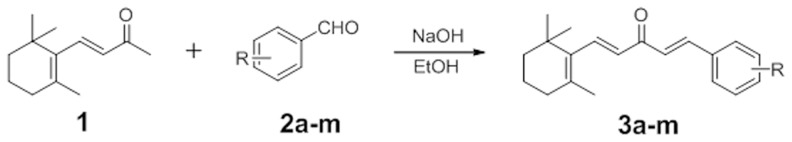
General route for the synthesis of 13 *β*-ionone and benzaldehyde analogues (3a−m). 1 =  *β*-ionone, 2a−m = 13 forms of benzaldehyde, 3a−m = 13 analogues of *β*-ionone and benzaldehyde. R is listed in Table 1.

Both *β*-ionone (0.88 g, 4.6 mmol) and benzene formaldehyde (**2a−m**, 6.0 mmol) were dissolved in ethanol (10 mL) in a round-bottom flask (50 mL) before 15 mL of a sodium hydrate solution (1 mmol/mL) was added dropwise. The reaction mixture was stirred at room temperature for 5 h, brought to pH 7.0 with 10% hydrochloric acid, and subsequently extracted with ether (50 mL ×2). The organic layer was dried with anhydrous Na_2_SO_4_, and the solvent was removed under reduced pressure. The residue was purified by column chromatography on silica gel using ethyl acetate-petroleum (60–90°C) at a ratio of 1∶13–1∶25 as the eluent to afford 13 analogues, which were designated **3a−m** (Figure 1) and R of the structures are illustrated in Table 1. They were characterized by melting point, IR, ^13^C NMR, ^1^H NMR and high-resolution mass spectrometry (HRMS). Melting points of chemicals were determined with an X-4 binocular microscope (Yuhua Instrument Co., Goyi, China) with thermometer. IR spectra were recorded on neat samples with a Nicolet 6700 FT-IR spectrometer (Thermo Fisher Scientific Inc., USA). ^13^C NMR and ^1^H NMR spectra were recorded with a Bruker Avance DPX300 spectrometer (Bruker-Spectrospin AG, Swiss). Chemical shifts were described in *δ* (ppm) relative to the signal of an internal standard (tetramethylsilane) and using CHCl_3_- *d*1 or DMSO-*d*6 as the solvent. Coupling constants were given in Hz. HRMS spectra were displayed under electron impact (150 eV) condition using a Bruker APEX IV spectrometer (Bruker Instruments Co. Ltd., USA).

**Table 1:** Physical properties, IR, HRMS and ^13^C NMR data for compounds 3a−m.

Compd	R	Mp(°C)	State	Yield (%)	IR (cm^−1^)	HRMS [M+H], (calcd)	^13^C NMR (75 MHz, *δ*, ppm)
3a	H	80–81	yellow crystal	61.3	2930, 2858, 1651, 1593, 1567, 1447, 1345	281.1905 (281.1900)	189.7, 144.3, 142.1, 137.9, 137.4, 134.8, 133.0, 130.5, 130.3, 127.1, 125.4, 40.8, 35.1, 34.7, 29.8, 22.8, 19.8
3b	4-F	69–70	yellow crystal	64.2	2933, 2865, 1667, 1598, 1572, 1455, 1339	299.1809 (299.1806)	189.9, 163.2, 144.2, 142.3, 137.8, 137.4, 132.1, 131.1, 130.4, 126.3, 117.2, 117.1, 116.8, 40.8, 35.1, 34.6, 29.8, 22.8, 22.4, 19.8
3c	4-Cl	87–89	yellow crystal	63.2	2930, 2859, 1654, 1597, 1567, 1410, 1352	315.1508 (315.1510)	189.7, 144.2, 142.0, 137.8, 137.4, 137.0, 134.4, 130.4, 130.3, 130.1, 127.0, 40.8, 35.1, 34.6, 29.8, 22.8, 19.8
3d	4-Br	95–97	yellow crystal	68.0	2928, 2858, 1654, 1596, 1564, 1489, 1350	359.1011 (359.1005)	190.0, 144.0, 143.6, 137.5, 135.9, 131.2, 130.5, 129.8, 129.2, 126.6, 40.8, 35.1, 34.6, 29.8, 22.8, 19.8
3e	4-Me	92–94	yellow crystal	76.2	2930, 2862, 1651, 1587, 1569, 1455, 1350	295.2055 (295.2056)	190.1, 143.8, 143.6, 141.6, 137.5, 137.2, 133.2, 130.6, 129.2, 125.8, 40.8, 35.1, 34.6, 29.8, 22.8, 22.4, 19.8
3f	4-Et	62–64	yellow crystal	32.8	2931, 2863, 1650, 1614, 1588, 1452, 1347	309.2215 (309.2213)	189.3, 147.1, 142.9, 136.5, 132.4, 130.0, 129.6, 128.5, 124.8, 39.8, 34.2, 33.7, 28.9, 21.9, 18.9, 15.4
3g	4-OMe	62–63	yellow crystal	78.5	2930, 2866, 1667, 1602, 1581, 1465, 1339	311.2009 (311.2006)	190.0, 162.4, 143.5, 143.4, 137.5, 137.0, 130.9, 130.7, 128.6, 124.5, 115.3, 56.3, 40.8, 35.1, 34.6, 29.8, 22.8, 19.9
3h	4-OEt	91–92	yellow crystal	42.3	2927, 2866, 1664, 1597, 1573, 1478, 1337	325.2163 (325.2162)	190.0, 161.8, 143.5, 137.5, 137.0, 132.9, 130.9, 130.7, 128.4, 124.4, 116.8, 115.6, 64.5, 40.8, 35.1, 34.6, 29.8, 22.7, 19.9, 15.6
3i	4-NO_2_	112–114	yellow crystal	68.7	2926, 2863, 1669, 1616, 1598, 1455, 1340	326.1752 (326.1751)	189.2, 149.4, 145.1, 142.1, 140.3, 139.0, 137.4, 130.1, 130.0, 129.7, 125.1, 40.8, 35.1, 34.8, 29.8, 22.8, 19.8
3j	3- Me	99–100	yellow crystal	53.2	2930, 2863, 1651, 1591, 1453, 1338	295.2062 (295.2056)	190.1, 143.9, 143.8, 139.5, 137.5, 137.4, 135.9, 132.0, 130.5, 129.8, 129.7, 126.5, 126.4, 40.8, 35.1, 34.6, 29.8, 22.8, 22.2, 19.8
3k	2- OMe	/	yellow oil	70.1	2930, 1647, 1622, 1592, 1562, 1487, 1350	311.2006 (311.2006)	190.8, 159.5, 137.5, 136.8, 132.7, 132.4, 131.1, 130.7, 129.7, 124.9, 121.6, 112.1, 56.4, 40.7, 34.5, 29.7, 22.7, 19.9
3l	3, 4-Me_2_	85–87	yellow crystal	75.1	2930, 2863, 1651, 1612, 1589, 1452, 1344	309.2217 (309.2213)	190.2, 143.9, 143.7, 140.4, 138.0, 137.5, 137.1, 133.6, 131.1, 130.6, 130.4, 126.8, 125.7, 40.8, 35.1, 34.6, 29.8, 22.8, 20.7, 20.6, 19.9
3m	2, 4-Cl_2_	109–110	yellow crystal	61.9	2938, 2865, 1667, 1610, 1565, 1468, 1384	349.1126 (349.1121)	189.7, 144.6, 138.1, 138.0, 137.4, 137.1, 136.7, 132.8, 130.9, 129.9, 129.6, 129.3, 128.4, 40.8, 35.1, 34.7, 29.8, 22.8, 19.8

### Stability of *β*-ionone, benzaldehyde and analogue (3 g)

**Figure 2:**
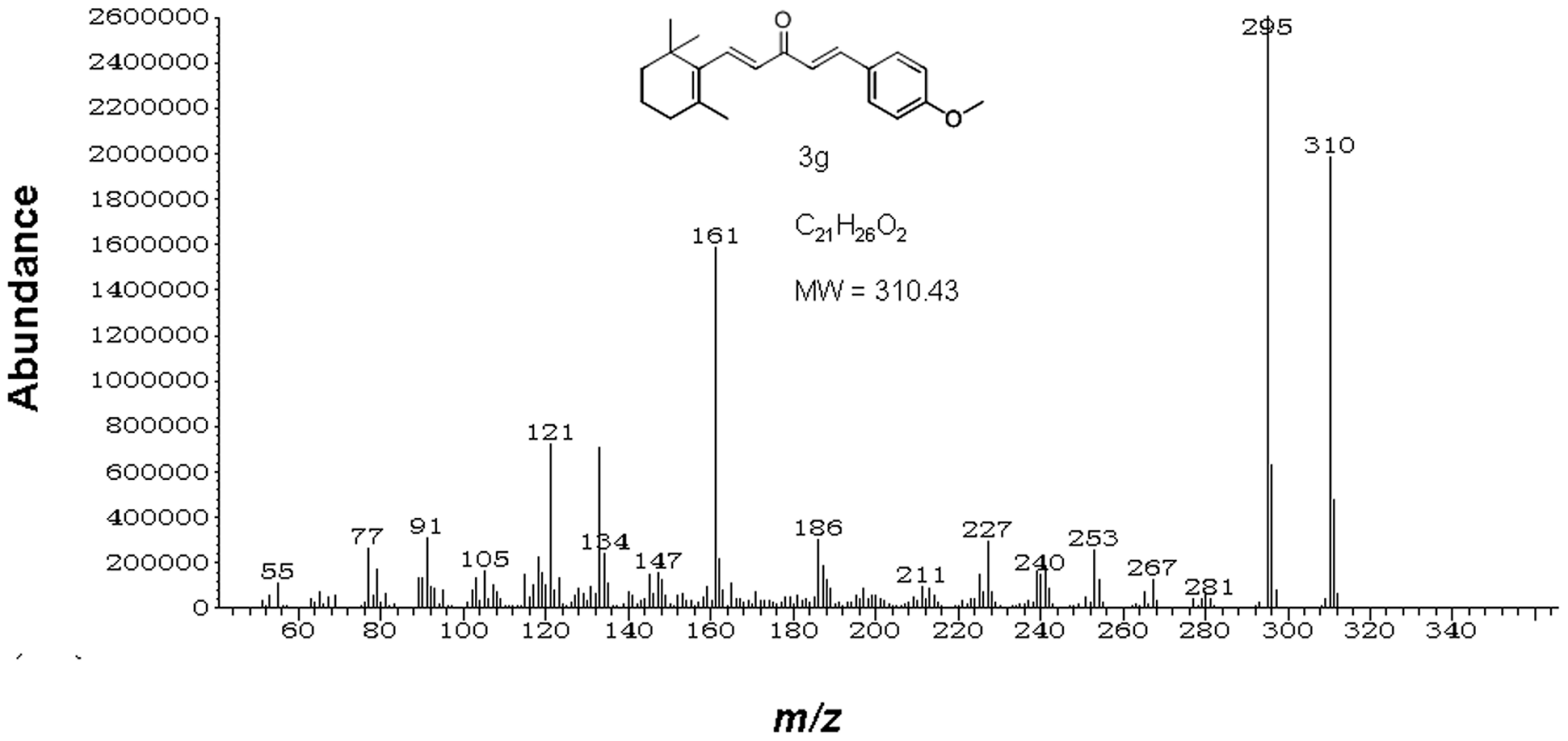
EI mass spectrum of analogue 3 g, also showing its structure, deduced molecular formula and molecular weight.

GC-MS analyses were used for testing the stability of *β*-ionone, benzaldehyde and one representative analogue, **3 g** (Figure 2), whose structure is quite similar to other synthesized analogues. GC-MS analyses were performed with a Thermo Trace GC Ultra gas chromatograph coupled to a Thermo ISQ mass spectrometer. The pure compounds (20 mg) were added into colorless, transparent vials (2-cm) individually. Then the vials were covered with 100 mesh gauzes and exposed to air and sunlight. 1 mg of each compound was sampled and stored in sealed brown, transparent vial every day. The sampling was last for seven days. Each compound was represented by three replicates. The GC was operated in splitless injection mode and fitted with a TG-5SILMS column (30 m×0.25 mm×0.25 µm). For *β*-ionone and benzaldehyde, firstly the oven was programmed from 40–130°C at 3°C/min after an initial delay of 1 min and held at 130°C for 1 min, and then the oven was programmed from 130–250°C at 10°C/min and held at 250°C for 5 min. For **3 g**, firstly the oven was programmed from 60–200°C at 10°C/min after an initial delay of 1 min and held at 200°C for 1 min, then the oven was programmed from 200–250°C at 10°C/min and held at 250°C for 10 min. Injector temperature was 250°C; MS quadrupole temperature was 150°C; MS source temperature was 250°C; and transfer line temperature was 250°C. The sampling and analyses were performed twice (in May 2013 and in September 2013) to confirm that whether the stability were consistent under different time horizons.

### Laboratory EAG experiment

The responses of *A. lucorum* females and males to the 13 analogues of *β*-ionone and benzaldehyde (**3a**−**m**) were measured by EAG experiment. The EAG responses of *A. lucorum* were recorded with Syntech GC/EAD interface temp controller TC-02 and stimulus controller CS-55 (Hilversum, The Netherlands). Laboratory EAG experiment was conducted to evaluate whether the test compounds stimulate *A. lucorum*. Five concentrations (0.01, 0.1, 1, 10, 100 µg/µL) in dichloromethane were checked in preliminary experiment. The concentration, 10 µg/µL, was suitable to each test compound. The compounds elicited relatively high EAG responses by *A. lucorum* at this concentration. Therefore we measured the EAG responses of the herbivore to chemicals only using the concentration, 10 µg/µL. And it was enough to evaluate whether the test compounds stimulate *A. lucorum*.

The test compounds were dissolved in distilled dichloromethane, and the solutions (20 µL, 10 µg/µL) were then added to a piece of folded filter paper (0.5 cm×4 cm). After evaporation for 30 s, the filter paper was inserted into a glass Pasteur pipette. The antennae of *A. lucorum* adults were excised and mounted between electrodes [30]. The stimuli were delivered to the antennae in a constant airstream of 150 mL/min at 30–40 s intervals, and the EAG signals were recorded. *β*-ionone and benzaldehyde were tested as positive controls. Dichloromethane and ethyl benzoate were also included as a background and standard stimulus, respectively, and these were applied before and after stimulation with each test compound. EAG responses were obtained from three replicate females and three replicate males per test compound.

The EAG responses of *A. lucorum* females and males to the samples of *β*-ionone, benzaldehyde, *β*-ionone + benzaldehyde and **3 g** exposed to air and sunlight and prepared in “**Stability of** ***β*** **-ionone, benzaldehyde and the analogue (3 g)**” were also measured.

The above laboratory EAG experiments were also performed twice (in May 2012 and in September 2013 for the first EAG experiment, and in July 2013 and in September 2013 for the second EAG experiment) to confirm that whether the responses of adult *A. lucorum* to the samples were consistent under different time horizons.

### Laboratory behavior experiment

Behavioural responses of adult *A. lucorum* to the pure and treated samples (exposed to air and sunlight for periods up to one and/or seven days) of *β*-ionone, benzaldehyde, *β*-ionone + benzaldehyde and **3 g** were investigated with a glass Y-tube olfactometer (2.8 cm uniform diameter, 21.8 cm main body length, and 18.8 cm branch length). An airflow (0.2 mL/min) was introduced into each arm of the olfactometer through glass stimulus chamber (an odour source adapter), attached to each of the two ending arms. In this way, two well-separated laminar air flows were generated in the olfactometer. As mentioned above, the concentration, 10 µg/µL in dichloromethane, was suitable to each test compound in the EAG experiment. The compounds elicited relatively high EAG responses by *A. lucorum* at this concentration. So here we used the same concentration, 10 µg/µL, for behavior assay. In each test 20 µL of dichloromethane solution of each chemical (10 µg/µL) was placed in the glass stimulus chamber of the “treatment” arm. As a control, 20 µL of dichloromethane was placed in the glass stimulus chamber of the “CK' arm of the olfactometer. Experiments were performed at room temperature. The olfactometer was washed with water and ethanol before each experiment. Adult *A. lucorum* was introduced at the bottom of the olfactometer individually and let free to walk. After 5 minutes, the *A. lucorum* in the treatment and control arms of the olfactometer was recorded. The insect that did not move and remained at the base of the Y tube was recorded as not reaction. Behavioral responses were obtained from 60 replicate females and 60 replicate males per test compound.

### Field experiment

An experiment was conducted in a 3-month-old alfalfa (*Medicago sativa* L.) field at the Xinxiang Experiment Station (35°18′ N, 113°54′ E) in Henan Province during 16–27 July 2012. The field had been tilled so that it was devoid of vegetation before alfalfa was planted; the alfalfa had not been sprayed with pesticide. For preparation of lures, the analogues of *β*-ionone and benzaldehyde (200 µL, 10 µg/µL in dichloromethane) were added into red rubber septa (Enoy Technology, Zhangzhou, China), and the dichloromethane was allowed to evaporate. The lures were positioned in the centers of white sticky cards (28 cm×22 cm), which were horizontally hung below ship-type traps (Enoy Technology, Zhangzhou, China) attached by wires to a bamboo stake; the traps were flush with the top of alfalfa plants (Figure S1). The trapping devices were placed ∼10 m apart, and were randomly assigned to contain one of the 13 analogues or the controls. Lures containing *β*-ionone, lures containing benzaldehyde and lures containing *β*-ionone + benzaldehyde prepared as above were used as positive controls. Red rubber septa without any compound were used as blank controls. Each compound or control was represented by three replicate traps. Sticky cards were replaced every 3 days, and the trapped *A. lucorum* were counted and sex was determined.

As with above method, another experiment was also conducted in this field during 15–22 July 2013. Lures containing one of *β*-ionone, benzaldehyde, *β*-ionone + benzaldehyde, **3 g** or blank controls were used. Sticky cards were replaced every day, and the trapped *A. lucorum* were counted and sex was determined.

### Statistical analysis

The relative EAG response value of *A. lucorum* antennae to each test compound was calculated using the following equation: Relative EAG response value  =  (EAG response value to the test compound – mean EAG response value to the background stimulus)/(mean EAG response value to the standard stimulus – mean EAG response value to the background stimulus). The mean relative EAG response values of female or male *A. lucorum* antennae to each test compound and to each positive control were compared using Student's *t*-tests. Student's *t*-tests were also used to compare the mean relative EAG response of female and male *A. lucorum* antennae to each test compound.

For the behavior research, the percent responses of *A. lucorum* were used for analysis and differences between “treated” and “CK” were compared with nonparametric tests followed by chi-square statistical.

For the field study, the cumulative numbers of *A. lucorum* collected in each sticky trap were used for analysis. One-way ANOVA was carried out for comparisons between the treatments.

All the statistic tests were conducted using SPSS (version 12.0).

## Results

Thirteen analogues (**3a−m**) were produced by the aldol reaction between *β*-ionone and benzene formaldehyde (**2a−m**) with minimal amounts of by-products (Fig. 1). This reaction was simple to perform, did not require dangerous conditions, and did not produce toxic substances. The 13 analogues were easily purified. Their identities were confirmed by IR, high-resolution mass spectrometry (HRMS), ^13^C NMR and ^1^H NMR. The physical properties, IR, HRMS and ^13^C NMR data for compounds **3a**–**m** are listed in **Table 1**. ^1^H NMR data for compounds **3a−m** are listed in **Table 2**.

**Table 2:** ^1^H NMR data for compounds 3a−m.

Compd	^1^H NMR (300 MHz, *δ*, ppm)
3a	1.11 (s, 6H, 5-Me, 5-Me), 1.48–1.52 (m, 2H, 4-H, 4-H), 1.61–1.67 (m, 2H, 3-H, 3-H), 1.83 (d, 3H, *J* = 0.7 Hz, 1-Me), 2.10 (t, 2H, *J* = 6.2 Hz, 2-H, 2-H), 6.48 (d, 1H, *J* = 16.1 Hz, 8-H), 7.00 (d, 1H, *J* = 15.9 Hz, 10-H), 7.39–7.42 (m, 3H, 14-H, 15-H, 16-H), 7.51 (d, 1H, *J* = 16.1 Hz, 7-H), 7.58–7.61 (m, 2H, 13-H, 17-H), 7.67 (d, 1H, *J* = 16.0 Hz, 11-H)
3b	1.11 (s, 6H, 5-Me, 5-Me), 1.48–1.52 (m, 2H, 4-H, 4-H), 1.60–1.65 (m, 2H, 3-H, 3-H), 1.83 (d, 3H, *J* = 0.7 Hz, 1-Me), 2.09 (d, 2H, *J* = 6.2 Hz, 2-H, 2-H), 6.46 (d, 1H, *J* = 16.1 Hz, 8-H), 6.93 (d, 1H, *J* = 15.9 Hz, 10-H), 7.09 (t, 2H, *J* = 8.7 Hz, 14-H, 16-H), 7.48–7.66 (m, 4H, 13-H, 17-H, 7-H, 11-H)
3c	1.11 (s, 6H, 5-Me, 5-Me), 1.48–1.51 (m, 2H, 4-H, 4-H), 1.61–1.67 (m, 2H, 3-H, 3-H), 1.83 (d, 3H, *J* = 0.7 Hz, 1-Me), 2.11 (t, 2H, *J* = 6.1 Hz, 2-H, 2-H), 6.46 (d, 1H, *J* = 16.1 Hz, 8-H), 6.97 (d, 1H, *J* = 15.9 Hz, 10-H), 7.36–7.54 (m, 5H, 7-H, 13-H, 14-H, 16-H, 17-H), 7.61 (d, 1H, *J* = 16.0 Hz, 11-H)
3d	1.11 (s, 6H, 5-Me, 5-Me), 1.48-1.52 (m, 2H, 4-H, 4-H), 1.61–1.68 (m, 2H, 3-H, 3-H), 1.83 (s, 3H, 1-Me), 2.10 (t, 2H, *J* = 6.2 Hz, 2-H, 2-H), 6.46 (d, 1H, *J* = 16.1 Hz, 8-H), 6.98 (d, 1H, *J* = 15.9 Hz, 10-H), 7.44-7.62 (m, 6H, 7-H, 11-H, 13-H, 14-H, 16-H, 17-H)
3e	1.11 (s, 6H, 5-Me, 5-Me), 1.48–1.52 (m, 2H, 4-H, 4-H), 1.60–1.68 (m, 2H, 3-H, 3-H), 1.82 (d, 3H, *J* = 0.3 Hz, 1-Me), 2.09 (t, 2H, *J* = 6.2 Hz, 2-H, 2-H), 2.38 (s, 3H, 15-Me), 6.47 (d, 1H, *J* = 16.1 Hz, 8-H), 6.96 (d, 1H, *J* = 15.9 Hz, 10-H), 7.12 (d, 1H, *J* = 8.0 Hz, 14-H), 7.50 (t, 2H, *J* = 6.8 Hz, 16-H, 7-H), 7.64 (d, 1H, *J* = 15.9 Hz, 11-H)
3f	1.11 (s, 6H, 5-Me, 5-Me), 1.25 (t, 3H, *J* = 7.6 Hz, 15-C-Me)1.48–1.52 (m, 2H, 4-H, 4-H), 1.63–1.66 (m, 2H, 3-H, 3-H), 1.83 (d, 3H, *J* = 0.6 Hz, 1-Me), 2.10 (t, 2H, *J* = 6.1 Hz, 2-H, 2-H), 2.68 (q, 2H, *J* = 7.6 Hz, 15-C-H, 15-C-H), 6.47 (d, 1H, *J* = 16.1 Hz, 8-H), 6.96 (d, 1H, *J* = 15.9 Hz, 10-H), 7.25 (d, 2H, *J* = 4.5 Hz, 14-H, 16-H), 7.50–7.53 (m, 3H, 7-H, 13-H, 17-H), 7.65 (d, 1H, *J* = 15.9 Hz, 11-H)
3g	1.11 (s, 6H, 5-Me, 5-Me), 1.48-1.52 (m, 2H, 4-H, 4-H), 1.60–1.66 (m, 2H, 3-H, 3-H), 1.82 (d, 3H, *J* = 0.7 Hz, 1-Me), 2.09 (t, 2H, *J* = 6.1 Hz, 2-H, 2-H), 3.85 (s, 3H, 15-O-Me), 6.47 (d, 1H, *J* = 16.0 Hz, 8-H), 6.86–6.93 (m, 3H, 14-H, 16-H, 10-H), 7.45–7.56 (m, 3H, 7-H, 13-H, 17-H), 7.64 (d, 1H, *J* = 15.9 Hz, 11-H)
3h	1.11 (s, 6H, 5-Me, 5-Me), 1.43 (t, 3H, *J* = 7.0 Hz, 15-O-C-Me)1.48-1.52 (m, 2H, 4-H, 4-H), 1.62–1.66 (m, 2H, 3-H, 3-H), 1.82 (s, 3H, 1-Me), 2.09 (t, 2H, *J* = 6.1 Hz, 2-H, 2-H), 4.07 (q, 2H, *J* = 7.0 Hz,15-O-C-H, 15-O-C-H), 6.46 (d, 1H, *J* = 16.0 Hz, 8-H), 6.85–6.92 (m, 3H, 10-H, 14-H, 16-H), 7.45–7.55 (m, 3H, 7-H, 13-H, 17-H), 7.64 (d, 1H, *J* = 15.9 Hz, 11-H)
3i	1.13 (s, 6H, 5-Me, 5-Me), 1.49-1.53 (m, 2H, 4-H, 4-H), 1.62–1.67 (m, 2H, 3-H, 3-H), 1.85 (d, 3H, *J* = 0.6 Hz, 1-Me), 2.12 (t, 2H, *J* = 6.2 Hz, 2-H, 2-H), 6.48 (d, 1H, *J* = 16.3 Hz, 8-H), 7.10 (d, 1H, *J* = 15.9 Hz, 10-H), 7.55–7.75 (m, 4H, 7-H, 11-H, 13-H, 17-H), 8.24–8.28 (m, 2H, 14-H, 16-H)
3j	1.12 (s, 6H, 5-Me, 5-Me), 1.48–1.52 (m, 2H, 4-H, 4-H), 1.63–1.67 (m, 2H, 3-H, 3-H), 1.83 (d, 3H, *J* = 0.7 Hz, 1-Me), 2.09 (t, 2H, *J* = 6.2 Hz, 2-H, 2-H), 2.39 (s, 3H, 14-Me), 6.48 (d, 1H, *J* = 16.1 Hz, 8-H), 6.98 (d, 1H, *J* = 16.0 Hz, 10-H), 7.19–7.32 (m, 2H, 13-H, 15-H), 7.40 (d, 2H, *J* = 5.8 Hz, 16-H, 17-H), 7.48 (d, 2H, *J* = 0.8 Hz, 16-H, 17-H), 7.53 (dd, 1H, *J* = 0.8 and 0.8 Hz, 7-H), 7.64 (d, 1H, *J* = 15.4 Hz, 11-H)
3k	1.11 (s, 6H, 5-Me, 5-Me), 1.48–1.52 (m, 2H, 4-H, 4-H), 1.60–1.68 (m, 2H, 3-H, 3-H), 1.82 (d, 3H, *J* = 0.7 Hz, 1-Me), 2.09 (t, 2H, *J* = 5.9 Hz, 2-H, 2-H), 3.89 (s, 3H, 13-O-Me), 6.50 (d, 1H, *J* = 16.1 Hz, 8-H), 6.91–7.09 (m, 3H, 10-H, 14-H, 16-H), 7.33–7.36 (m, 1H, 15-H), 7.45 (dd, 1H, *J* = 0.7 and 0.8 Hz, 7-H), 7.59 (q, 1H, *J* = 3.1 Hz, 17-H), 7.99 (d, 1H, *J* = 16.2 Hz, 11-H)
3l	1.11 (s, 6H, 5-Me, 5-Me), 1.48–1.52 (m, 2H, 4-H, 4-H), 1.60–1.66 (m, 2H, 3-H, 3-H), 1.83 (s, 3H, 1-Me), 2.10 (t, 2H, *J* = 6.1 Hz, 2-H, 2-H), 2.29 (s, 6H, 14-Me, 15-Me), 6.49 (d, 1H, *J* = 16.0 Hz, 8-H), 6.95 (d, 1H, *J* = 15.9 Hz, 10-H), 7.16 (d, 1H, *J* = 7.6 Hz, 13-H), 7.34 (q, 2H, *J* = 4.3 Hz, 16-H, 17-H), 7.49 (d, 1H, *J* = 15.7 Hz, 7-H), 7.63 (d, 1H, *J* = 15.9 Hz, 11-H)
3m	1.12 (s, 6H, 5-Me, 5-Me), 1.48–1.52 (m, 2H, 4-H, 4-H), 1.63–1.67 (m, 2H, 3-H, 3-H), 1.83 (d, 3H, *J* = 0.7 Hz, 1-Me), 2.11 (t, 2H, *J* = 6.2 Hz, 2-H, 2-H), 6.50 (d, 1H, *J* = 16.1 Hz, 8-H), 6.93 (d, 1H, *J* = 16.0 Hz, 10-H), 7.27–7.30 (m, 1H, 16-H), 7.45–7.55 (m, 2H, 7-H, 14-H), 7.62 (d, 1H, *J* = 8.5 Hz, 17-H), 7.97 (d, 1H, *J* = 16.0 Hz, 11-H)

**Figure 3:**
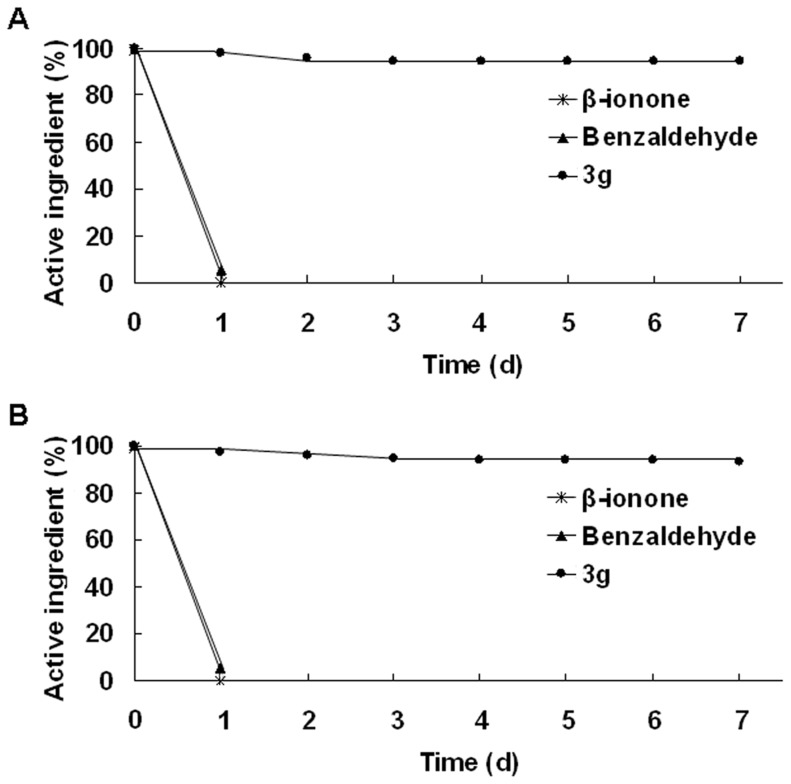
Active ingredient content of *β*-ionone, benzaldehyde and 3 g on different day after exposing them to air and sunlight for a period up to seven days. (A) Stability tests performed in May 2013, (B) Stability tests performed in September 2013.

The stability of *β*-ionone, benzaldehyde and one representative analogue, **3 g** (Fig. 2), were checked after exposing them to air and sunlight for a period up to seven days. In such conditions, the stability tests performed in May 2013 indicated that samples of pure *β*-ionone degraded completely and samples of pure benzaldehyde declined to 5.5% a.i. on the first day, whereas samples of pure **3 g** declined to 94.3% a.i. on the seventh day (Fig. 3A and Fig. S2). And the stability tests performed in September 2013 showed a similar result (Fig. 3B). The experiments confirmed that the stabilities of the samples were consistent under different time horizons and **3 g** was much more stable in the environment than *β*-ionone and benzaldehyde.

A previous study showed that *β*-ionone and benzaldehyde elicited EAG responses from adult *A. lucorum* antennae, suggesting that the compounds were recognized as signals by *A. lucorum* adults [29]. To test the attractiveness of the analogues to *A. lucorum*, we first measured the EAG responses elicited by the analogues from adult *A. lucorum*. In the case of the experiment performed in May 2012, the mean EAG response value to the background stimulus, dichloromethane, was −35.74±21.10 µV for *A. lucorum* females and −39.08±19.80 µV for males. The mean response values to the standard stimulus, ethyl benzoate (20 µL, 10 µg/µL), was −80.03±62.55 µV for females and −94. 8±50.15 µV for males, which were significantly higher than to the background (both P<0.001). The difference between females and males in their responses to the standard stimulus was not significant (P = 0.083).

**Figure 4:**
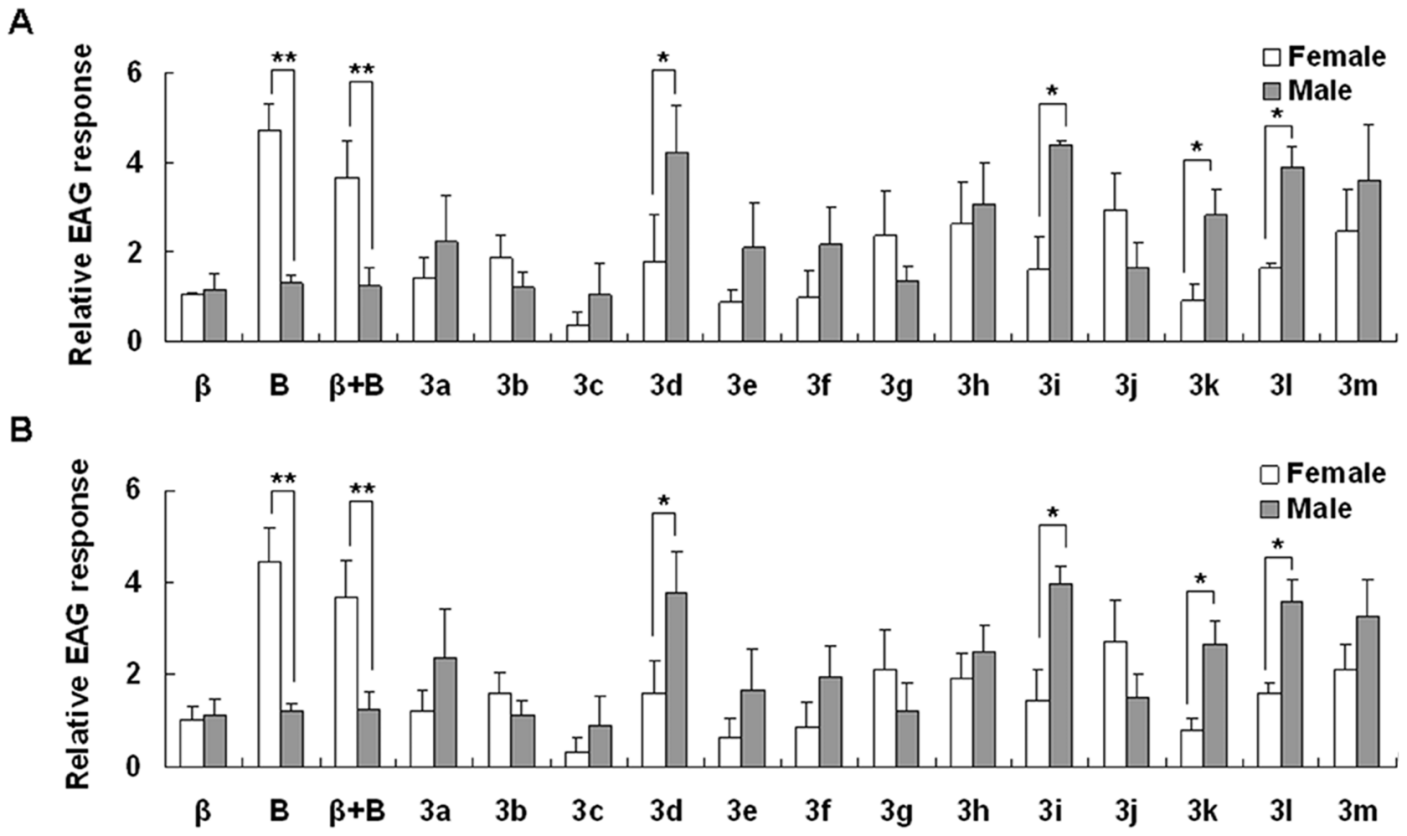
Relative EAG responses (mean ± SD) of female and male *A. lucorum* to synthetic analogues of *β*-ionone and benzaldehyde. (A) EAG experiment performed in May 2012, (B) EAG experiment performed in September 2013. *β* =  *β*-ionone and B =  benzaldehyde. Asterisks indicate significant differences in EAG responses between female and male antennae: * P<0.05, ** P<0.01.

*β*-ionone, benzaldehyde and *β*-ionone + benzaldehyde were included as positive controls in our EAG response experiment. The mean relative EAG response values to *β*-ionone (***β***) was 1.05±0.02 for female *A. lucorum* and 1.16±0.34 for males, and the difference between females and males was not significant (P = 0.632). In contrast, the response to benzaldehyde (**B**) was stronger for females (4.74±0.59) than for males (1.30±0.17) (P<0.01), and the response to *β*-ionone + benzaldehyde (***β***+**B**) was also stronger for females (3.69±0.79) than for males (1.23±0.39) (P<0.01) (Fig. 4A).

Among the 13 synthesized compounds, only three (**3b**, **3g**, and **3j**) elicited stronger EAG responses from female *A. lucorum* antennae than from male antennae; for all other compounds, male antennae exhibited stronger responses than female antennae (student's *t*-tests P<0.05) (Fig. 4A). Compounds **3 h** and **3 m** elicited stronger responses from both female and male antennae than other analogues (Fig. 4A).

The EAG responses of female antennae were stronger to compounds **3h**, **3j**, and **3m** than to the positive control *β*-ionone (P<0.05), while responses of female antennae were weaker to all 13 analogues than to the positive control benzaldehyde and *β*-ionone + benzaldehyde (P<0.05). The responses of male antennae were stronger to **3d**, **3h**, **3i**, **3k**, **3l**, and **3m** than to *β*-ionone, benzaldehyde and *β*-ionone + benzaldehyde (P<0.05).

The EAG experiment performed in September 2013 showed a similar pattern as above (Fig. 4B). Our results demonstrate that most analogues synthesized in this study elicit responses from *A. lucorum* and the responses of adult *A. lucorum* to the samples were consistent under different time horizons.

**Figure 5:**
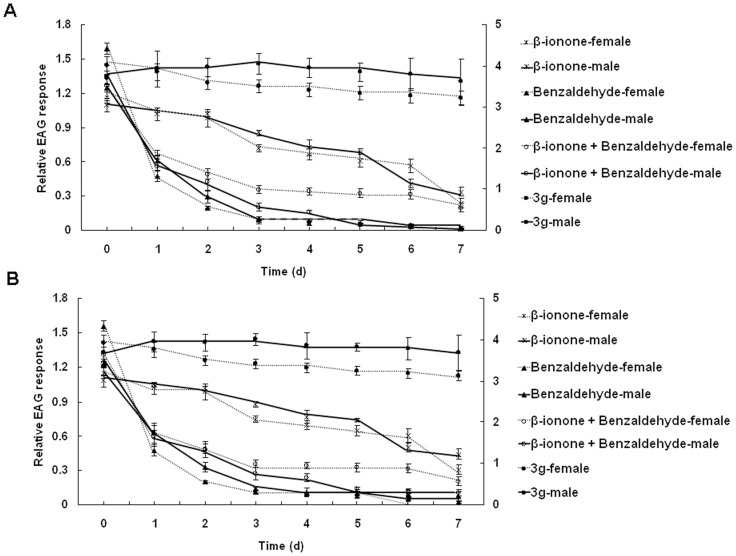
Relative EAG responses (mean ± SE) of female and male *A. lucorum* to *β*-ionone, benzaldehyde, *β*-ionone + benzaldehyde and 3 g. (A) EAG experiment performed in July 2013, (B) EAG experiment performed in September 2013. Secondary axis in the chart showed relative EAG responses of female *A. lucorum* to benzaldehyde and to *β*-ionone + benzaldehyde.

Samples of *β*-ionone, benzaldehyde, *β*-ionone + benzaldehyde and **3g** that were exposed to air and sunlight for different periods were tested for their EAG responses with antennae of adult female and male *A. lucorum*. In the case of the experiment performed in July 2013, all samples of *β*-ionone, benzaldehyde and **3g** elicited EAG responses from female and male *A. lucorum* antennae (Fig. 5A). The relative EAG response values to *β*-ionone, benzaldehyde and *β*-ionone + benzaldehyde decreased day by day. On the contrary, the relative EAG response values to **3g** changed very little over seven days. The EAG experiments were performed again in September 2013 and showed a similar result (Fig. 5B).

**Figure 6:**
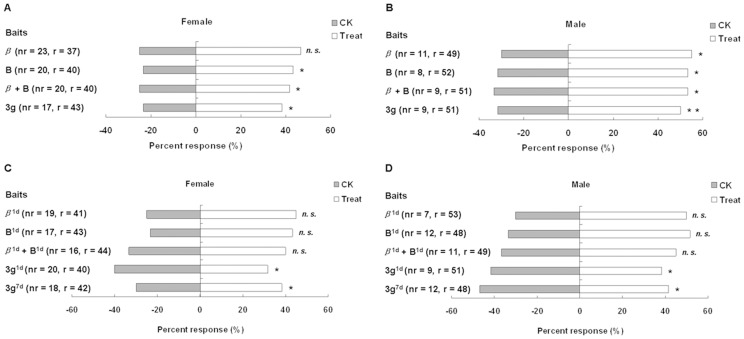
Choices of adult *A. lucorum* in the Y-tube olfactometer. (A) female towards pure *β*-ionone, benzaldehyde, *β*-ionone + benzaldehyde and 3 g, (B) male towards pure *β*-ionone, benzaldehyde, *β*-ionone + benzaldehyde and 3 g, (C) female towards *β*-ionone^1d^, benzaldehyde^1d^, *β*-ionone^1d^ + benzaldehyde^1d^, 3g^1d^ and 3g^7d^, (D) male towards *β*-ionone^1d^, benzaldehyde^1d^, *β*-ionone^1d^ + benzaldehyde^1d^, 3g^1d^ and 3g^7d^. The bars represent the percentage of tested insects that made a particular choice. The asterisks with the choice bars indicate significant preferences. *P<0.05, **P<0.01, n.s.  =  not significant, nr  =  not reacting, r =  reacting. Superscript characters of compounds represent the periods that the chemicals left exposing to air and sunlight.

In behavior experiment, *A. lucorum* showed preferences for the pure *β*-ionone, benzaldehyde, *β*-ionone + benzaldehyde and **3 g** when tested against solvent dichloromethane (CK). For female *A. lucorum*, the differences were only statistically significant for pure benzaldehyde, *β*-ionone + benzaldehyde and **3 g** (Fig. 6A), but for male *A. lucorum*, the differences were statistically significant for all compounds (Fig. 6B). The differential attractiveness between odours of *β*-ionone^1d^ (pure *β*-ionone left exposing to air and sunlight for one day, the same below) and CK was not pronounced in the experiments where they were offered together as choices. The results obtained in benzaldehyde^1d^–CK and (*β*-ionone^1d^ + benzaldehyde^1d^)–CK were similar to *β*-ionone^1d^–CK foregoing. Whereas, female and male *A. lucorum* showed a significant preference for **3g** ^1d^ and **3g** ^7d^ when they were offered next to CK respectively (Fig. 6C and 6D). Our results demonstrated that **3 g** was attractive to adult *A. lucorum* and its attractiveness persisted longer than *β*-ionone, benzaldehyde and *β*-ionone + benzaldehyde.

**Figure 7:**
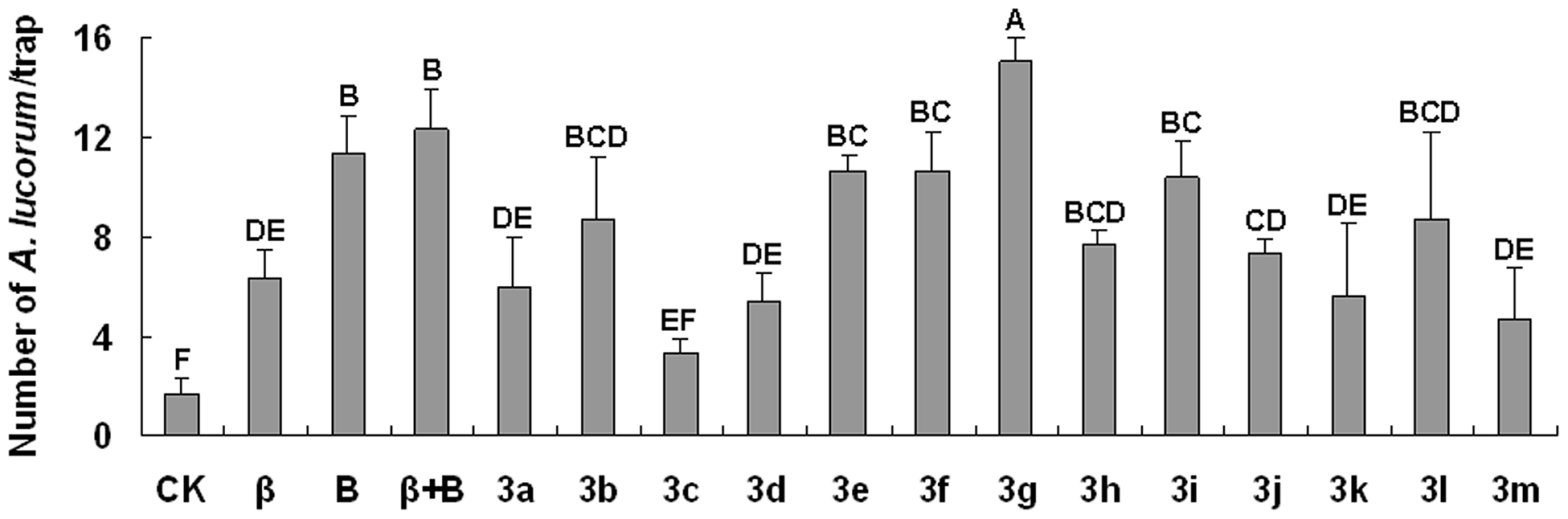
Number of *A. lucorum* captured in sticky traps (mean ± SD per trap) baited with synthetic analogues of *β*-ionone and benzaldehyde during 16–27 July 2012. CK  =  blank control, *β* =  *β*-ionone, and B =  benzaldehyde, *β*+B =  *β*-ionone + benzaldehyde. Means with the same letter are not significantly different.

We then determined whether the analogues of *β*-ionone and benzaldehyde could attract *A. lucorum* in an alfalfa field. The results showed that the volatiles *β*-ionone, benzaldehyde and *β*-ionone + benzaldehyde (positive controls) attracted significantly more *A. lucorum* (females and males) than dichloromethane (blank control, **CK**). The number of *A. lucorum* in traps containing analogue lures varied with the analogue. Compounds **3a**, **3c**, **3d**, **3j**, **3k**, and **3m** trapped a moderate number of *A. lucorum*, while **3b**, **3e**, **3f**, **3g**, **3h**, **3i**, and **3l** trapped a high number *A. lucorum*. In particular, **3g** trapped significantly more *A. lucorum* than positive control (Fig. 7).

The compounds that were most attractive in the field were not the same compounds that elicited the strongest EAG responses in the laboratory. For example, **3d**, **3k**, and **3m** elicited strong EAG responses in the laboratory but did not result in the trapping of a high number of *A. lucorum* in the field. Laboratory EAG experiment was conducted in an unnatural environment. The odours in the experimental area, interference from instrument and human activities may influence the result of EAG experiment. In addition, field experiment was carried out in a natural environment. Insect physiological condition, environmental condition and other factors can affect the efficiency of the tested compounds used in the fields [11]. Therefore EAG response values may only represent whether the test compounds stimulate insects, but not reflect positive correlation with the insects behavior [31]–[33].

**Figure 8:**
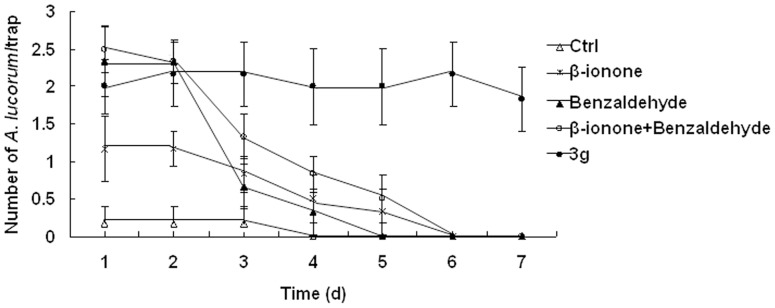
Number of *A. lucorum* captured in sticky traps (mean ± SE per trap) baited with *β*-ionone, benzaldehyde, *β*-ionone + benzaldehyde and 3 g from different day during 15–22 July 2013.

We also determined whether *β*-ionone, benzaldehyde, *β*-ionone + benzaldehyde or **3 g** attracted *A. lucorum* constantly. The result showed that *β*-ionone, benzaldehyde and *β*-ionone + benzaldehyde attracted significantly more *A. lucorum* than dichloromethane (blank control, **Ctrl**) in the first two days and then attracted very little *A. lucorum* in the last five days. However, the analogue **3 g** trapped sustained and balanced numbers of *A. lucorum* during seven days (Fig. 8). This indicated that **3 g** possessed persistent attractiveness to *A. lucorum*.

## Discussion

Plants synthesize and release volatile organic compounds. These signals can be detected by phytophagous insects via olfactory sensilla on the antennae and used to locate hosts and avoid non-host plants [34]–[36]. Plant volatiles have been artificially synthesized and successfully used as pest attractants in IPM. For example, methyl anthranilate has been used as an attractant for the thrips *Thrips hawaiiensis* Morgan and *Thrips coloratus* Schmutz [17]. A blend of *cis*-3-hexene acetate, linalol, and methyl jasmonate was shown to be attractive to the Colorado potato beetle, *Leptinotarsa decemlineata* (Say) [18].

*β*-ionone and benzaldehyde are common plant volatiles released by many kinds of crops [20]–[23]. Although a recent study indicated that *β*-ionone and benzaldehyde can alter the behavior of *A. lucorum* adults under laboratory conditions [29], they could not be efficiently used for pest control in the field because of their low stability and insufficient attractiveness.

So far there has been no publish about plant volatile analogues synthesis and their use. In the current study, we try to explore novel ecological approach to control pest based on synthetic plant volatile analogues. We first hypothesized and synthesized analogues of *β*-ionone and benzaldehyde that combined moieties of the chemicals in order to produce a compound with increased stability and enhanced attractiveness to *A. lucorum* adults.

In this study, the analogues synthesized were demonstrated to have high stability compared to the original plant volatiles *β*-ionone or benzaldehyde. The laboratory EAG experiment showed that most of the analogues elicited responses from *A. lucorum* adult antennae. The laboratory behavior experiment displayed that analogue **3 g** was attractive to *A. lucorum* and its attractiveness persisted longer than the original plant volatiles. The field experiment indicated that most of the analogues were attractive to *A. lucorum* adults. The high stability and persistent attractiveness of analogue **3 g** in particular make it suitable for field use and potential as an attractant. The results support our hypothesis that designing and synthesizing analogues by combining the bioactive components of *β*-ionone and benzaldehyde would contribute to increasing stability and enhancing attractiveness.

Now it's necessary to increase the number of studies like this to design, synthesize plant volatile analogues and evaluate their bioactivities to insect. Once this is accomplished, there is the possibility of supporting it as a widely-accepted ecological approach that may extend its use in pest control in the field.

## Supporting Information

Figure S1 **One of the traps deployed in the field experiment.**
(TIF)

Figure S2 **Total ionic chromatogram and mass spectrum of** ***β*** **-ionone, benzaldehyde and 3 g, treated by leaving them exposing to air and sunlight for periods.**
(TIF)
