# Supplementary figures and images for: Plant Volatile Analogues Strengthen Attractiveness to Insect
Source: PLoS One. 2014 Jun 9;9(6):e99142. doi: 10.1371/journal.pone.0099142 (PMC4049612; doi:10.1371/journal.pone.0099142)

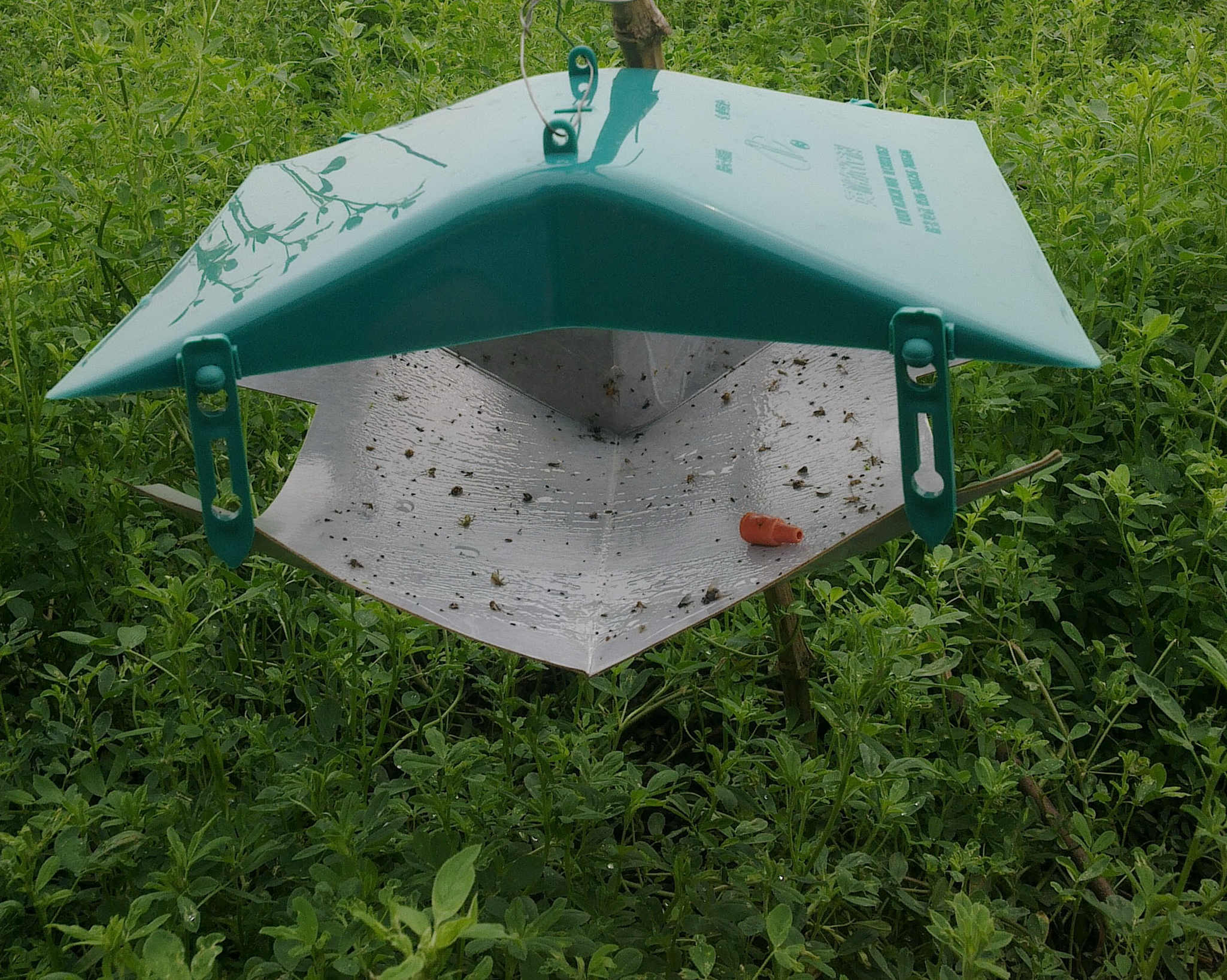

Supplement: Figure S1 — One of the traps deployed in the field experiment. (TIF) [file pone.0099142.s001.tif]

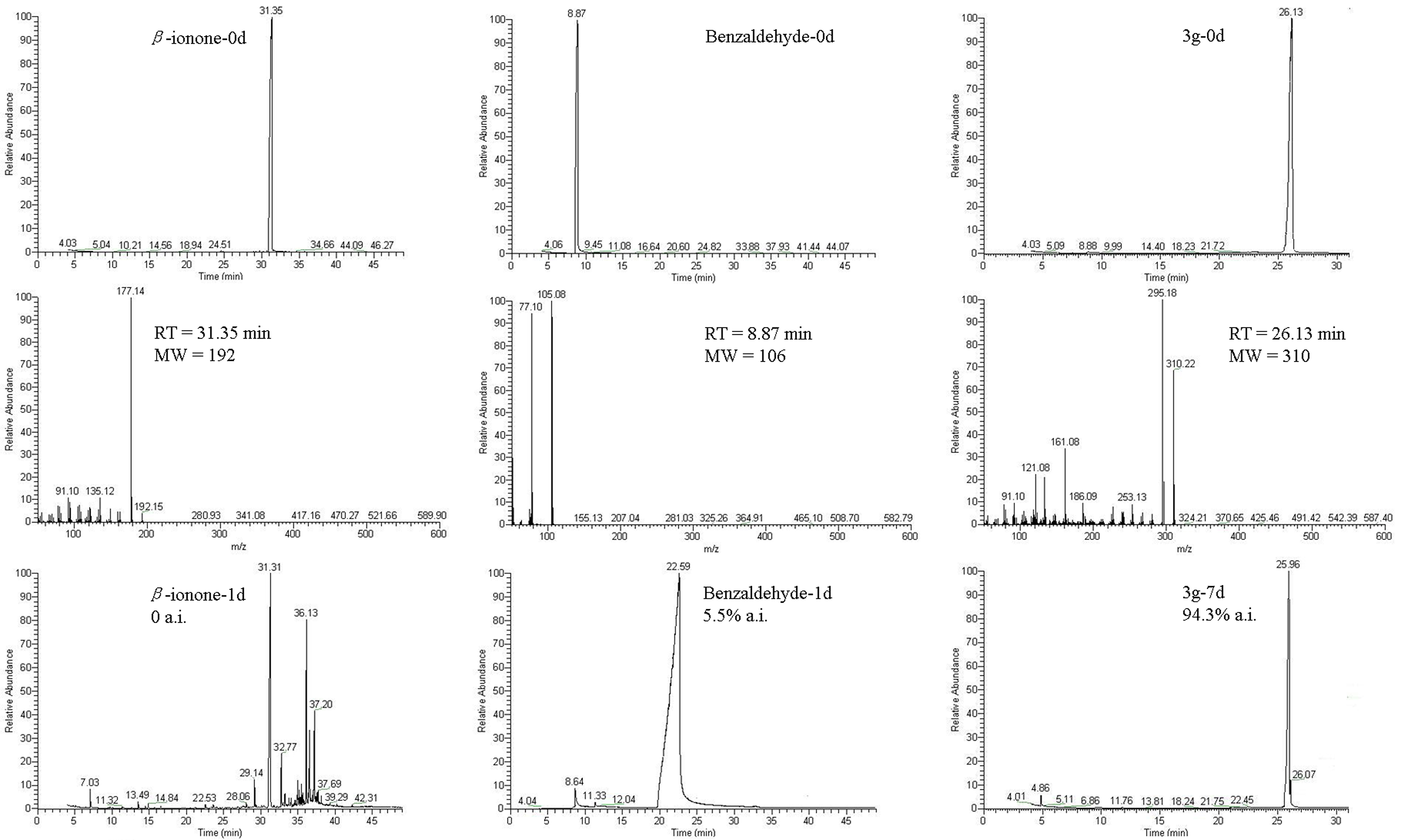

Supplement: Figure S2 — Total ionic chromatogram and mass spectrum of β -ionone, benzaldehyde and 3 g, treated by leaving them exposing to air and sunlight for periods. (TIF) [file pone.0099142.s002.tif]
